# Does Early Recruitment Predict Greater Physical Performance in Academy Soccer Players?

**DOI:** 10.3390/sports6040108

**Published:** 2018-09-30

**Authors:** Maxime Hertzog, Darren J. Paul, George P. Nassis, Joao R. Silva

**Affiliations:** 1 National Sports Medicine Programme, Excellence in Football Project, Aspetar-Orthopedic and Sports Medicine Hospital, Sports City, 29222 Doha, Qatar; Darren.Paul@aspetar.com (D.J.P.); georgenassis@gmail.com (G.P.N.); Joao.silva@aspetar.com (J.R.S.); 2 Center of Research, Education, Innovation and Intervention in Sport (CIFI2D), Porto 4000, Portugal

**Keywords:** long-term, team sport, jump, speed, aerobic

## Abstract

The purpose of this longitudinal study was to investigate whether recruitment status influences neuromuscular and endurance performances in academy soccer players over a 2-year training period (from Under-16 to Under-18). Thirty-seven male soccer players from an elite academy were selected and divided in two cohorts according to their recruitment status: Early Recruitment group (ER; *n* = 16), training and competing for the academy since Under-14 and Under-15 age groups, and; Late Recruitment group (LR; *n* = 21) included in the academy training process at Under-16. Squat (SJ) and countermovement jump with (CMJwA) and without arms swing (CMJ), 10-m sprint time, and Vam-Eval test (MAV) were performed in three successive occasions always pre-season (Under-16, Under-17 and Under-18 age groups, T1, T2, and T3 respectively). A two-way (recruitment status × time) analysis of variance with repeated measurements was performed as well as the magnitude of difference using both effect size and magnitude-based inferences. There was no difference between ER and LR for MAV, 10 m-sprint, and SJ from T1 to T3. However, LR players presented non-significant small and possibly greater improvement in CMJ (ES = 0.4) and CMJwA (ES = 0.4) than ER players at T2. These data indicate that early recruitment is not likely to result in greater physical performance improvement at the age of 18.

## 1. Introduction

Talent identification and development is considered paramount to ensure long-term economic sportive success and sustainability of professional soccer clubs [1,2]. The traditional approach to talent identification and selection has been linked to subjective and preconceived image of the ideal player [3]. Research addressing the acquisition and development of soccer expertise has focused on key performance characteristics related to task constraints (specificity and amount of soccer practice), performers constraints (e.g., physiological factors) and environmental constraints (e.g., sociocultural influences) [4]. Nevertheless, due to the different qualities associated with performance in youth soccer (e.g., physical and psychological), a holistic multidisciplinary approach has been recommended to coaches and scouts [1,4,5,6,7,8]. An important issue is that successful performance in soccer is not related on one standard set of skills, but can be achieved in unique ways through different combinations of abilities [9]. Thus the clubs’ objective is to recruit and nurture players with the intention to play first team soccer. The premise is likely that of providing an environment (e.g., high-level coaching and training conditions) that allows their ability to be nurtured. In doing so, players will have earlier exposure to technical and tactical as well as structured physical conditioning [10]. Intuitively, it is clear to see the future competitive and financial gains associated with early recruitment into a professional soccer academy [1,2,6]. Being so, talent identification and selection is especially important for smaller clubs due to their inferior’s financial resources [2].

While earlier recruitment may be preferred, players will often enter at different stages in the soccer specialization process [2,4,6]. Recent longitudinal observations show a higher rate of physical development in academy, compared to non-academy soccer players, independent of the initial performance level of the child and change in maturation over the same period of time [10]. For instance, Wrigley et al. [10] reported significant improvement of 8.6% and 22.4% in 10 m sprint and countermovement jump without arm swing respectively, over a 3-year soccer academy follow-up (from 12 to 16-years old). Sander and colleagues [11] showed significant improvement of 1.9% in 10 m sprint performance in elite soccer players after a 2-year follow-up (from 17 to 19-years old). The premise being that earlier recruitment will equate to greater training exposure and enhanced physical characteristics, however, this has not yet been investigated. The majority of the long-term studies compared changes in physical performances between players having substantial difference in training load mainly due to additional trainings (e.g., strength, speed, power, endurance) [11] or to their soccer status (e.g., academy vs. non-academy, elite, recreational) [10].

Therefore, the purpose of the study was to investigate whether recruitment status (early vs. late into the academy influences neuromuscular and endurance performances in youth soccer players over a 2-year soccer academy training program. 

## 2. Materials and Methods

### 2.1. Experimental Approach to the Problem

The primary objective of this longitudinal study was to investigate the effect of players’ recruitment strategy (early vs. late) into an elite soccer academy on jump ability, sprint time and incremental field running test performances during a 2-year training program. Data collection was conducted over a 5-year period (2009–2013). As a result of the Academy selection process (players’ had attended all testing sessions throughout the entire follow-up) and recent injury history, a final sample of 37 youth soccer players was included in the analysis. Players’ birth-date distribution was computed. The first testing protocol was conducted at Under-16 (U16) age group. The second and the third testing protocol were performed at Under-17 (U17) and Under-18 (U18) age groups respectively. The players were assessed annually, each time at the beginning of the pre-season (August). The difference between groups baseline performance and the two following seasons were chosen as the primary outcome.

### 2.2. Participants 

After applying the selection criteria, players where divided in two cohorts according to their recruitment status: Early Recruitment group (ER; *n =* 16; age 15.4 ± 0.1 years, height 170 ± 7 cm, body mass 61.7 ± 5.0 kg); training and competing already for the academy at Under-14 (U14) and Under-15 (U15) age groups and Late Recruitment group (LR; *n =* 21; age 15.2 ± 0.3 years, height 173 ± 7 cm, body mass 63.7 ± 6.8 kg); included in the academy training process at U16 (start of the observation period). U16 competed in the regional championship while Under-17 (U17) and Under-18 (U18) teams competed in the national championship. All teams followed the same training methodology during the follow-up period with an average of 10 h of training and competitive play per week (4–5 soccer training sessions, 1 strength training session, 1 conditioning training session, 1 game per week). The generic training and competition plan during the different periodization periods is presented in Table 1. In summary, pre-season period comprised, on average, 5–6 weeks duration and the competitive season lasted on average 30 weeks. While approval for the study was obtained from the technical director of the club’s academy, the data was obtained as part of the normal testing and training program in which the player performance was evaluated over the course of training during the competitive season [12]. Therefore, appropriate ethics committee clearance was not required; however, the study conformed to the Code of Ethics of the World Medical Association (Declaration of Helsinki).

### 2.3. Procedures

Players were always tested in the morning (9:30 AM) during the second day of the pre-season to avoid any diurnal change. No intense physical activity was performed in the first day back. All the tests were performed outdoor on a synthetic field with players wearing soccer boots. The testing was preceded by a 15-min standardized warm-up consisting of athletic drills, jumps, dynamic stretching and progressive and maximal accelerations followed by 3 min of rest. The players completed the physical tests in the same order in all the assessment moments. The testing order was: as follow: squat jump (SJ), countermovement jump (CMJ), countermovement jump with free arm use (CMJwA), 10-m sprint and an incremental field running test (Vam-eval). All players were familiar with the tests procedures, which were part of their previous testing routine in lower age group categories (LR) or during regional or national selections (ER).

### 2.4. Jump Ability 

During each SJ trial, players started from a 90° squat down position and with hands on hips and then extended maximally their knees. Countermovement jumps were performed in two ways: with the hands on the hips (CMJ) and with free arms use (CMJwA). During each CMJ and CMJwA trial, participants started from a standing position, squatted down and then extended maximally their knees in one continuous movement. Players were asked to perform three maximal trials for each jump test with 15 s rest between trials and minimum 2 min rest between tests to ensure adequate recovery. Jump performance was recorded using a validated optical system (Optojump^®^; Microgate, Bolzano, Italy) with an accuracy of 1 per 1000 s [13]. The highest vertical jump (cm) was considered for analysis. The reliability of SJ and CMJ has shown to be high (ICC; SJ = 0.96; CMJ = 0.97) in junior soccer players [14,15].

### 2.5. Sprint Times

Acceleration (10-m linear sprint time, Acc) was recorded using photocell gates (Brower Timing Systems, Salt Lake City, UT, USA) with an accuracy of 0.01 s. The timing gates were placed at a height of 80 centimetres measured from the ground to the top of the cells. Players began each sprint when ready from a standing start with their front foot 0.5-m behind the first timing gate and were instructed to sprint to their maximum ability over the 10-m distance. Two trials with two minutes of walking and passive recovery were performed. The best sprint time was used for analysis. The reliability of Acc (10-m sprint) has shown to be high (ICC = 0.87) in academy soccer players [16].

### 2.6. Incremental Field Running Test

To end the test session, the players performed a continuous multistage field test (Vam-eval) on a 200-m outdoor circular running track (synthetic field) demarcated with ten cones placed every 20 m as a reference. The Vam-eval is a modified version of the aerobic fitness test initially proposed by the University of Montreal (UM-TT) to approach maximal aerobic velocity (MAV) [17]. The only difference between the two tests is the distance between the cones placed along the athletic track (i.e., 20-m (Vam-eval) vs. 50-m (UM-TT)), which renders the Vam-eval easier to administer to young soccer players [18]. The test starts with an initial running speed of 8.5 km·h^−1^ and increases by 0.5 km·h^−1^ every minute until exhaustion. Players adjusted their running speed to the cones placed at 20-m intervals according to auditory signal. During the test, testers and coaches verbally encouraged the players. The test was terminated when the player could no longer maintain the required running speed dictated by the audio beep, for three consecutive occasions (cones). The average velocity of the last minute completed was recorded as the players’ MAV (km·h^−1^). If the last stage was not fully completed, 0.1 km·h^−1^ was attributed for 15 s additional running time, 0.3 km·h^−1^ for 30 s and 0.4 km·h^−1^ for 45 s. MAV was calculated as the last completed velocity in km·h^−1^ and the additional velocity attributed for each 15 s covered during the uncompleted stage [16]. The reliability of Vam-eval to assess MAS was reported in a similar population (ICC = 0.77) [16].

### 2.7. Statistical Analyses

A chi-square test was run to check birth-date distributions against those expected based on 1:1:1:1 ratio. Expected birth-date distributions were estimated to be 25% for each quarter of the year [19]. All performance data were first log-transformed to reduce bias arising from non-uniformity error. The normal distribution of the data was checked with the Shapiro–Wilk test. A two-way (recruitment status × time) analysis of variance (ANOVA) with repeated measurements was performed. Sidak adjustments for multiple post hoc comparisons were applied when a significant F-value was found. The level of significance was set at *p* < 0.05. The data was analyzed using the IBM^®^ SPSS^®^ Statistics V22.0 software (NY, USA). Additionally, within and between-conditions changes (percentage of performance change) in MAV, SJ, CMJ and CMJwA were calculated from a specifically designed Excel spreadsheet, using pooled standard deviations [20]. Differences were expressed as 90% confidence limits (CL) and as probabilities that the true effect was substantially greater or smaller than the smallest worthwhile change (between-subjects SD/5) [20]. Threshold values for standardized differences were trivial (<0.2), small (0.2–0.6), moderate (0.6–1.2), large (1.2–2.0) and very large (>2.0) [21]. These probabilities were used to make a qualitative probabilistic mechanistic inference about the true effect. The scale was as follows: 25−75%, possible; 75−95%, likely; 95−99%, very likely; >99%, almost certain. If the 90% confidence interval (CI) overlapped small positive and negative values, the magnitude was deemed unclear; otherwise, that magnitude was deemed to be the observed magnitude [21]. Data in text and tables are presented as mean with standard deviations (SD).

## 3. Results

Baseline physical performance measures of both early and late groups are presented in Table 2. Chronological age between ER and LR groups was significantly moderate and very likely different at baseline. Moreover, we observed a difference (*p* < 0.001) in birth-date distribution between the quarters of the year in ER (X^2^_3_ = 24.00, N = 16) while no difference (*p =* 0.11) was observed in LR group (X^2^_3_ = 5.85, N = 21). ER players born early in the year were highly represented in the first (Q1 = 75%) than the subsequent quarter (Q2 = 25%, Q3 = 0% & Q4 = 0%). Similar trend was observed in LR group (Q1 = 48%, Q2 = 14%, Q3 = 19% & Q4 = 19%). 

Only MAV was moderate and likely (with chances for greater/similar/lower performance of 89/10/1, respectively) greater in the ER group compared to LR group at baseline. No clear (all difference rated as unclear) difference between groups existed in 10-m sprint and jump ability at baseline (Table 2). From U16 to U18, both groups presented moderate and likely to very likely changes on MAV (Table 3). Over the same period, the 10-m linear sprint performances indicated large and to almost certainly decrement of time in both groups. LR players presented large and almost certainly improvements in SJ, CMJ, CMJwA from T1 to T3 while ER players improvements were only rated as moderate and very likely to almost certainly.

There is no clear and no significant performance change difference between ER and LR in MAV, 10 m-sprint and SJ from T1 to T3. However, LR players presented non-significant small and possibly greater improvement in CMJ (71/26/03) and CMJwA (70/26/4) than ER players at T2 (Table 3).

## 4. Discussion

The results of this study showed that early recruitment does not result in greater physical performance improvement compared to late recruitment in youth soccer players.

The changes in physical performance ranged from 4.2% to 5.4% in MAV, 3.8% to 2.7% in 10 m sprint and 12.7% to 20.9% in jump ability, in our sample. Additionally, these gains were similar in early and late recruiters. Other studies have shown improvement in 10 m sprint ranging from 3.9% in U15 elite players over a 2-year period [11], to 9.4% in 11–15-year-old academy players over a 3-year period [10,11]. Additionally, the latest study reported an 18.3% improvement in CMJ performance [10].

We observed that early and late recruitment player’s performance was enhanced by 0.6 vs. 0.7 km/h^−1^ and 0.07 vs. 0.09 s in MAV and 10 m sprint, respectively. Performance in SJ, CMJ and CMJwA tests improved by 5.0 vs. 6.0 cm, 4.5 vs. 6.6 cm, 5.1 vs. 7.2 cm for ER and LR respectively. These gains are comparable to those reported in other studies with soccer academy players [10,11]. Improvement of 0.04 to 0.08 s in 10 m sprint in U-17 elite soccer players after a 2-year follow-up have been reported elsewhere [11]. A recent longitudinal study conducted with youth soccer players reported an improvement of 0.13 s in 10 m sprint and 6.4 cm in countermovement jump over a 3-year follow-up [10]. These previous findings demonstrate that long-term player development programs, such as those implemented in professional soccer academies, accelerate the rate of physical development.

We observed that early recruitment players’ endurance performance was higher than players involved at a later stage in the soccer academy. In fact, as we suggested previously, higher exposure to systematic training per se may contribute to enhance physical performance. Interestingly, a recent study reported that short speed performance is generally more resistant to training enhancement compared to other physical capabilities [22]. In the present investigation, only MAV performance was likely higher in ER compared to LR group at baseline (T1). Notably, while both groups likely (ER) to very likely (LR) improved their MAV over the 2-year training period, LR players were not able to close this small and likely lower MAV performance gap with ER players. Their substantially higher running speed to exhaustion may be due to the training background rather than difference in genotypic backgrounds. In fact, high-intensity interval training was performed consistently for ER players throughout U14-U15 ages within the academy.

Overall, ER does not provide a higher magnitude of improvement compared to LR group when comparable training methodology and content are performed. Conversely, LR players presented small and possibly greater gains in CMJ (4.2 ± 5.7%) over the entire follow-up. Although key functional capabilities (e.g., endurance and muscle power) seem to discriminate players already selected and exposed to systematic training [4] our findings indicate that early recruitment may not necessarily elicit greater leg power or endurance capacity. Interestingly, it may also suggest that a latter start of training of these skills may not be a limiting factor to achieve a higher level of performance. Evidence from other sports demonstrates that early specialization is not the only way to reach high-level of expertise [4]. Variability in the individual responses to endurance [23,24,25] and strength trainings [24,26] are well established. Inter-individual responses to a given training are potentially due to the variance in training volume and/or intensity [24]. Possibly, the concurrent training of endurance and strength within the training program may have influenced the observed results. Soccer can be considered a concurrent sport by nature due to the associated endurance and neuromuscular-related demands triggered by the training of the other relevant constructs of performance (technical and tactical) [27]. Moreover, the periodization model applied may influence performance adaptations [28,29]. Further studies should investigate the effect of player’s specialization time (early vs. late recruitment) and training periodization paradigm (block training approach vs. traditional training design) on physical and physiological development. In addition, players’ capacity to deal with daily adversity in trainings and competitions could interfere with the expression of performance. In fact, sport psychology research reported that training and competing under negative (i.e., anxiety, stress and anger) and positive (i.e., enjoyment, satisfaction and happiness) emotions are part of the construct of athlete’s performance [24]. 

Identification and selection of young soccer players are highly influenced by chronological age and indicators which are age-dependent [19,30,31,32,33]. In the present study, ER players born early in the year were highly represented in the first (Q1 = 75%) than the subsequent quarter (Q2 = 25%, Q3 = 0% & Q4 = 0%). The trend that players born early in the year might be more mature, stronger and faster than their younger counterparts may partly explain this disproportionate representation in youth soccer player’s selection at an early age. Unfortunately, we have no way of assessing the validity of these previous assumptions in our study. Nevertheless, the relative age effect (RAE) and it’s relationships with maturation, anthropometry and physical performance were previously examined across a representative sample of English professional youth academies [33]. Lovell and colleagues identified small advantages in body size and speed performance in relatively older players at the entry point to English soccer developmental programmes. Thus, findings support the recent opinion [16] questioning the interest to use physical performance measure at early age as a reliable tool to predict future success of youth soccer players. In Germany, professional players seem to emerge from repeated procedure of selection and de-selection through youth and childhood rather than at early selection [34].

We acknowledge the potentially different biological maturation status between the groups at the baseline. Additionally, the relatively small sample size, which included only one professional academy, may limit the generalization of the results. Finally, it is worth highlighting that our findings are only applicable to physical characteristics with endurance and power in particular. 

## 5. Conclusions

This study showed that early recruitment does not result in greater physical performance improvement compared to late recruitment in youth soccer players. Our results question the current belief that early recruitment may ensure a greater performance development in relevant soccer physical performance parameters. It does not imply that we should not recruit players early as soccer performance is multidimensional and is affected not only by the physical fitness elements. These findings may help coaches and decision-makers to address the right priorities along long-term player development pathway.

## Figures and Tables

**Table 1:** Generic pre-season and in-season weekly cycle organization.

Period of Year	Time of Day	Monday	Tuesday	Wednesday	Thursday	Friday	Saturday	Sunday
Pre-season	a.m.	Conditioning	Conditioning	Soccer	Conditioning	Rest	Rest	Rest
	p.m.	Soccer	Soccer	Rest	Soccer	Soccer	Training or Game	Rest
In-season	a.m.	Rest or Recovery	Soccer	Conditioning	Soccer	Rest	Soccer	Rest
	p.m.	Rest or Recovery	Strength	Rest	Rest	Soccer	Rest	Game

**Table 2:** Age and physical performance at baseline (mean ± SD).

Performance	Early Recruitment (*n* = 16)	Late Recruitment (*n* = 21)	Difference ES
Chronological age (years)	15.4 ± 0.13	15.2 ± 0.3	−0.8 * ^###^
MAV (km·h^−1^)	16.5 ± 0.9	16.0 ± 1.0	−0.6 ^##^
10 m (s)	1.88 ± 0.06	1.90 ± 0.06	0.3
SJ (cm)	33.0 ± 3.7	33.5 ± 4.3	0.1
CMJ (cm)	34.7 ± 4.1	34.4 ± 4.8	−0.1
CMJwA (cm)	41.2 ± 5.4	41.5 ± 6.8	0.0

* *p* < 0.01 between recruitment status. The number of “#” refers to a possible (#), likely (##) and very likely (###) difference. ES—effect size, MAV-maximal aerobic velocity, SJ—Squat jump, CMJ—countermovement jump, CMJwA—countermovement jump without arm-swing.

**Table 3:** Within and between groups (early vs. late) differences on performance changes across the 2 years training (mean ± SD).

Performance Metric	U16-U17			U17-U18			U16-U18		
	Early	Late	ES	Early	Late	ES	Early	Late	ES
∆ MAV (km·h^−1^)	1.8 ± 4.1	2.7 ± 3.6 **	0.1	2.4 ± 4.4	3.0 ± 3.6 *	0.0	4.2 ± 3.7	5.4 ± 3.4 ***	0.1
∆ 10 m (s)	−1.2 ± 1.8	−1.9 ± 1.9	−0.1	−2.7 ± 2.0 *	−3.4 ± 2.0 **	−0.1	−3.8 ± 2.1 **	−4.9 ± 1.7 ***	−0.3
∆ SJ (cm)	8.8 ± 7.6	9.8 ± 5.8 *	0.1	5.5 ± 8.3	6.9 ± 6.4	0.0	14.8 ± 8.2 **	16.2 ± 7.4 ***	0.1
∆ CMJ (cm)	4.1 ± 6.9	11.0 ± 6.5 *	0.4 ^#^	9.0 ± 6.2 *	9.2 ± 5.3 *	0.1	12.8 ± 6.8 **	20.9 ± 7.7 ***	0.2
∆ CMJwA (cm)	1.0 ± 8.0	10.2 ± 7.9	0.4 ^#^	11.5 ± 6.3 *	9.8 ± 6.6 *	−0.1	12.7 ± 7.4 **	20.2 ± 8.9 *	0.0

∆—Performance change in percentage, * *p* < 0.05; ** *p* < 0.01; ***p < 0.001 in performance change. “#” denotes a possible difference in between groups performance changes. ES—effect size, MAV—maximal aerobic velocity, SJ—Squat jump, CMJ—countermovement jump, CMJwA—countermovement jump without arm-swing.
